# Evaluation of Phosphene Shifts During Eye Movements to Enhance Safe Visual Assistance for Visually Impaired Individuals

**DOI:** 10.3390/bioengineering12030281

**Published:** 2025-03-11

**Authors:** Manami Kanamaru, Keita Tanaka, Eiji Kamioka

**Affiliations:** 1 Division of Electronic Information and Biomedical Engineering, Tokyo Denki University, Saitama 350-0394, Japan; ktanaka@mail.dendai.ac.jp; 2 Shibaura Institute of Technology, Graduate School of Engineering and Science, Tokyo 135-8548, Japan; kamioka@shibaura-it.ac.jp

**Keywords:** phosphene, visually impaired, Hess chart

## Abstract

Hands-free visual assistive devices that consider the safety of the visually impaired have been researched, but many of them interfere with other senses, such as hearing. Therefore, phosphenes have been researched as a method of presenting visual information. Phosphenes are flashes that are recognized by electrical stimulation, and the presentation position can be adjusted by the electrode arrangement. However, it has been reported that the presentation position changes significantly when the eyeballs are moved as far left and right as possible. As a walking assistive device for the visually impaired, the fluctuation of the presentation position of phosphenes may cause safety problems. This study used the Hess test to verify the positional fluctuation of phosphenes associated with eye movement and compared it with eye movement during walking to discuss safety. As a result, the range of the change in the presentation position of phosphenes was significantly large when subjects moved their eyes to the peripheral vision, the same as in the previous study. On the other hand, the presentation position of phosphenes did not change significantly within the range of eye movement during walking (±15 deg). Our results suggest that there is the possibility that serious safety issues will not happen with assistive devices for the visually impaired who use phosphenes.

## 1. Introduction

As visually impaired people cannot obtain visual information about the outside world, they use visual assistive devices such as white canes. However, a white cane cannot always be used with one hand, which could be dangerous in an emergency. Therefore, hands-free visual assistive devices have been researched; however, many devices in research studies block other senses, such as hearing [1,2]. Therefore, phosphenes have been researched to provide visual information despite visual impairment [3,4].

Phosphenes are flashes of light recognized by electrically stimulating the eyeball and visual cortex. The presentation position of phosphenes can be controlled by adjusting the position of the electrodes on the face [3,4,5]. In terms of stimulating the eyeball, the stimulated area has been discussed in several studies [3,6]. Laakso reported that the electric current flows to the retinae along the eye socket using electric simulation when the phosphenes are observed [6]. However, their hypothesis on the electric current flow is inconsistent with the real observed position of phosphenes reported in several studies. If the electric current flows to the retinae along the eye socket, the phosphene should be observed on the left side of vision when the temporal right eyeball is stimulated. However, several studies have reported that the phosphene was observed on the right side, when the temporal right eyeball was stimulated. Moreover, a previous study has reported that, when the eyeball is moved to the left and right for peripheral vision, the phosphene presentation position was changed because the visual retina on the anterior side may also be exposed and stimulated directly [3]. The visual retina exposed by eye movements should be stimulated if the electric current flows along the eye socket; however, the observed phosphene position was extremely different from the reported phosphene presentation position [3,5]. Therefore, a previous study suggested that the cornea and sclera, which are the eyeball surfaces exposed to the anterior side, and the retina, which is located at the opposite pole of the stimulated area, were stimulated [3]. Therefore, the phosphene presentation position can be controlled by calibrating the electrode arrangement on the face to stimulate the intended eyeball surface area.

However, when using phosphenes as a walking assistive device for the visually impaired, the position of the phosphenes changes significantly due to eye movement, which is a safety issue for the walking assistive device. Although the electric current does not flow along the eye socket and stimulate the retina, the depth to which facial skin and eyeballs are stimulated by the electric current has not been clarified.

Therefore, in this study, the Hess test was used to gradually move the eyeball and verify how the position of the phosphenes changes in this case. The related works about eye movement during walking were also investigated and the results were compared to discuss the safety of visual assistive devices that use phosphenes. This study clarifies that eye movements during walking do not affect the presentation position of phosphenes. Moreover, this study contributes to clarifying the stimulation depth of the electrical current around the eye.

The rest of the paper is organized as follows: Section 2 discusses related studies and background knowledge, and the evaluation methods are presented in Section 3. Section 4 describes the evaluation results. Section 5 discusses the results, and the conclusions of this study are presented in Section 6.

## 2. Related Studies and Background Knowledge

### 2.1. Eyeballs’ Internal Structure and a Change in Phosphene Presentation During Eye Movement

This section discusses the internal structure of the eyeball and the position of the phosphenes as they change with eye movement.

**Figure 1:**
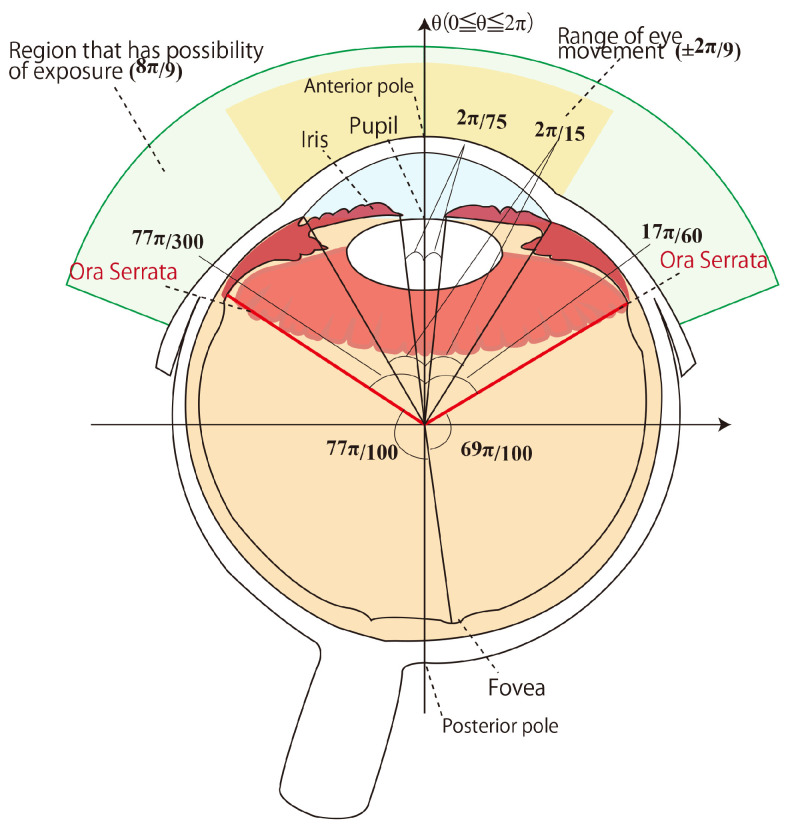
Sectional diagram of the right eyeball.

Figure 1 shows a horizontal cross-section of a human right eyeball. The human eyeball is spherical and has a diameter of 24 mm [7]. The cornea, located on the eyeball’s sur-face, protrudes from the sphere. The eyeball’s anterior pole is set at 0 rad, and the azimuth angle is set in the 0 to 2π rad clockwise range, as shown in Figure 1. The diameters of the pupil and iris are 2 mm and 10 mm, respectively; so, the central angles of the areas are 4π/75 rad and 4π/15 rad. The white part of the eye outside the iris is called the sclera. Most of the inside of the eyeball is covered by the retina, which is divided into the visual retina, where photoreceptors are located, and the blind retina, where photoreceptors are not located. The visual retina occupies the dorsal 3/4 of the inside of the eyeball, and the blind retina occupies the remaining 1/4 [8] (pp. 2761–2762). The Ora Serrata exists at the boundary between the visual retina and the blind retina. Since the human eyeball inverts images with the lens, in the case of the right eyeball, information from the right visual field is processed by the nasal retina, and the temporal retina processes information from the left visual field. However, when obtaining information from the left visual field from the right eyeball, the nose prevents observing the edge of the peripheral field on the left side. In addition, visual information from the center of human vision is projected onto the fovea. The fovea is located 1 mm from the posterior pole of the eyeball. From this positional relationship, as shown in Figure 1, the central angle of the area where the nasal retina receives information from the right visual field is 77π/100 rad, and the central angle of the area where the temporal retina receives information from the left visual field is 69π/100 rad. The distance from the anterior pole to the Ora Serrata also differs between the nasal and temporal sides, with the central angle being 77π/100 rad on the nasal side and 69π/100 rad on the temporal side [9,10].

In a previous study that reported on the change in the presentation position of phosphenes due to eye movement [3], it was assumed that 8π/9 rad of the anterior surface of the eyeball could be exposed to the anterior side. When the eyeball is directed to the center, 4π/9 rad of the anterior surface of the eyeball is exposed to the anterior side [11]. This information was considered based on the anatomical positional relationship of the bulbar conjunctiva and the conjunctival lid. The eye movement required for the Ora Serrata to be exposed to the anterior side was 77π/300 rad and 17π/60 rad for the left and right sides, respectively.

In a previous study, subjects were asked to draw and report the location of phosphenes when they moved their eyes to the center, left, and right as far as possible while their head was fixed [3]. Ten subjects participated, and six electrode arrangements were used: three electrode arrangements to stimulate the right eye and three electrodes arrangements to stimulate the left eye were used. The stimulation current used was an alternating current that was reported to be capable of observing phosphenes, and the stimulation values were 0.3 mA or 0.8 mA, 10 V, and 10 Hz. As a result, it was reported that, when the eyes were moved to the left and right as far as possible, the visual retina exposed on the anterior side was stimulated by the eye movement, and phosphenes were presented in the visual field controlled by the exposed visual retina. It was also reported that, when the eyes were directed toward the center, the visual retina was not exposed on the anterior side, and phosphenes were presented in the visual field controlled by the retina located opposite to the stimulated eye surface.

However, previous studies have not discussed how much eye movement significantly changes the presentation position of phosphenes. In this study, the presentation of phosphenes in the intended position is considered necessary for the safety of walking assistive devices for visually impaired people. Therefore, it is necessary to verify how much the presentation position of phosphenes changes when eye movements are performed gradually.

### 2.2. Eye Movements During Walking

This section mainly mentions related studies that reported eye movements during walking. It has been reported that the general saccades that occur naturally in sighted people are within 15 deg [12]. Saccades are eye movements that rapidly shift the fixation point and are performed to view a target object with the fovea [8] (pp. 1328–1329). A related study [12], which used electrooculography (EOG) to investigate the distribution of saccade amplitudes during long-term walking in three sighted subjects, found that most saccades were less than 15 deg.

A related study has also been reported in which visually impaired people participated as subjects [13]. The related study aimed to develop a head-mounted display device to assist patients with peripheral vision defects due to diseases such as retinitis pigmentosa in collecting information necessary for walking [13]. When the peripheral vision is impaired, the head and eyes must be linked to collect information in the impaired area. Therefore, three sighted people and five retinitis pigmentosa patients participated and how they moved their heads and eyes during walking was measured. Only the retinitis pigmentosa patients used a visual refraction device and a white cane during the experiment. All subjects walked independently for a total of more than 30 min. Their experiment was conducted indoors and outdoors to measure eye movements, avoiding weather conditions that make it difficult to observe the pupils.

It was found that the horizontal eye movement was within 20 deg in sighted people and within 13 deg in retinitis pigmentosa patients. This experimental result indicates that the eye movement of retinitis pigmentosa patients is about two-thirds of a normal visual field. It suggests that, even if the visual field is lost, visual information is collected not only by eye movements but also by head movements.

There are no reports of eye movement during walking in blind patients who cannot receive any visual information. However, it can be assumed that the eye movement during walking in sighted people and retinitis pigmentosa patients, who generate saccades in response to visual information from the outside world, is larger than that of blind patients. Therefore, the reports of related works [12,13] could be used for comparison when discussing the safety of walking assistive devices that change the presentation position of phosphenes due to eye movements during walking, which is the focus of this study.

## 3. The Presentation Position of Phosphenes Changes with Eye Movements

This section describes the method to verify how much the presentation position of phosphenes changes when eye movements are gradually performed, as well as the evaluation method.

### 3.1. Verification of Experimental Conditions and Methods

**Figure 2:**
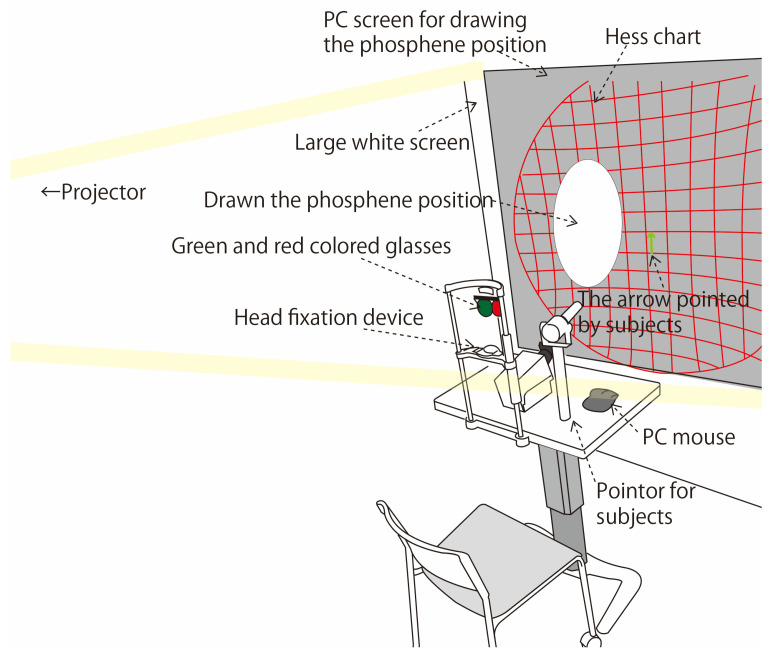
Verification experiment environment.

**Figure 3:**
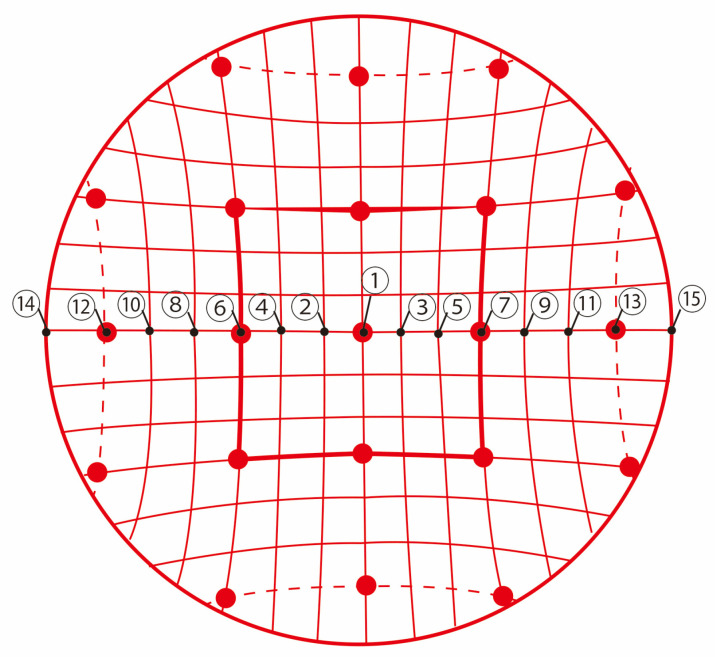
The order and position of eye movements in the verification experiment.

Figure 2 shows the environment of the verification experiment. The subjects who participated in the verification experiment were seated on a chair in a dark room. First, the Hess test was used to check whether the subjects had problems with eye movements, such as strabismus. The Hess chart projector used in the Hess test was made by Handaya Co., Ltd. (Tokyo, Japan) [14]. A 1.0 m projector was used for the Hess test, and a white projector screen was placed in front of the subjects. Then, the subjects gazed at the indicated point on the Hess chart while the phosphenes were presented, and the presentation position of the phosphenes at that time was drawn using a mouse. There were 15 points in the horizontal direction on the Hess chart, corresponding to a visual field of ±30 deg. In a previous study [3], the limit of eye movement was set at ±2π/9 rad (approximately ±40 deg). The range of eye movement instructed in the previous study [3], ±40 deg, exceeds the angle of eye movement on the Hess chart, ±30 deg. Therefore, in this experiment, to reproduce the conditions of eye movement in the previous study, the participants were instructed to move their eyes to the right and left as far as possible, just as in the previous study [3]. Figure 3 shows the positions and order of the fixation points on the Hess chart in the verification experiment. Odd fixation points indicate where the eyes were moved to the right, and even fixation points indicate where the eyes were moved to the left. Subjects pointed with the green pointer used in the Hess test at the fixation point indicated by the red pointer.

When drawing the presentation position of the phosphenes, the PC screen projected by the projector behind the subjects and overlaid with the Hess chart was operated with the mouse at hand. CLIP STUDIO PAINT was used for drawing, and the circle tool in the functions of the application was used [15]. The size of the layer for drawing the phosphenes was set to 2200 × 1800 pixels, which is enough to cover the horizontal direction of the Hess chart.

**Figure 4:**
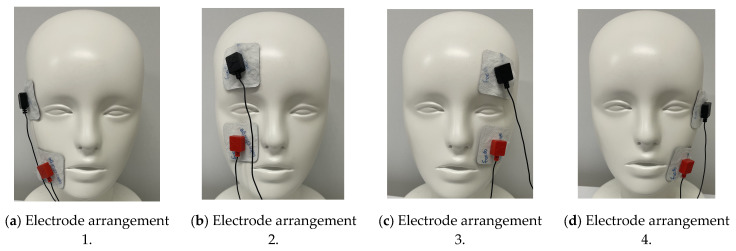
Electrode arrangements for the verification experiment. (**a**) Electrode arrangement 1 presents phosphenes to the right side of the visual field. (**b**) Electrode arrangement 2 stimulates the right eyeball and present phosphenes at the center of the visual field. (**c**) Electrode arrangement 3 stimulates the left eyeball and present phosphenes at the center of the visual field. (**d**) Electrode arrangement 4 presents phosphenes to the left side of the visual field.

The electrode arrangements used for presenting the phosphenes were based on four patterns from previous studies, as shown in Figure 4 [4]. Each arrangement served a distinct purpose: electrode arrangement 1 presented phosphenes to the right side of the visual field, electrode arrangement 2 presented phosphenes at the center of the visual field by stimulating the right eye, electrode arrangement 3 presented phosphenes at the center of the visual field by stimulating the left eye, and electrode arrangement 4 presented phosphenes to the left side of the visual field. The stimulation device used in the experiment was foc.us, which can use tACS (transcranial alternating current stimulation) [16]. Before the verification experiment, the stimulation current value was examined in the range from 0.3 mA to 1.0 mA according to the skin condition of the subject. The voltage and frequency values were 10 V and 10 Hz, according to the previous study [3].

In the Hess test, the movement of the right and left eyes can be limited by viewing a Hess chart through glasses in which one eye is red and the other is green. In this verification experiment, three patterns were verified: when the right eye is red, when the left eye is red, and when the glasses are removed.

The subjects were 10 sighted men and women aged in their 20s. This verification experiment was conducted in accordance with the Declaration of Helsinki and approved by the Institutional Review Board of Tokyo Denki University. Informed consent was obtained from all subjects before the verification experiment was conducted.

### 3.2. Evaluation Methods

**Figure 5:**
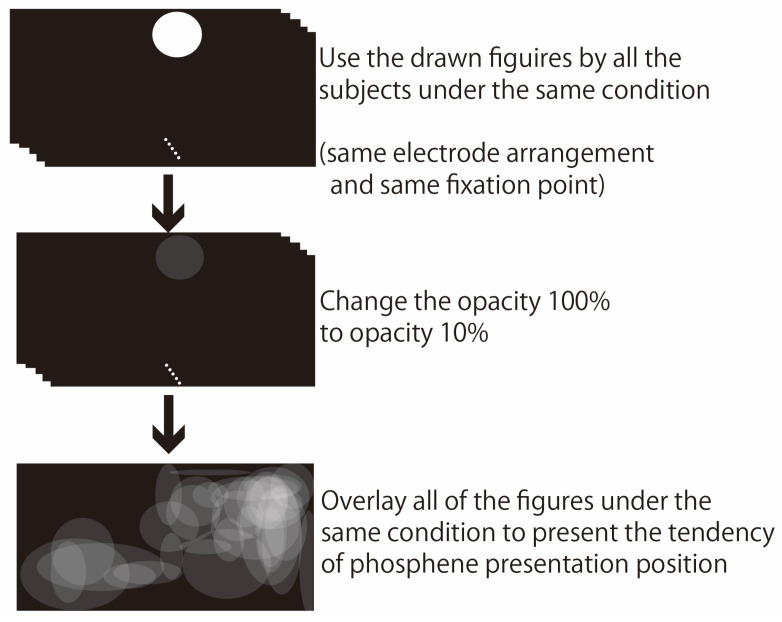
Qualitative evaluation methods.

In this verification experiment, the presentation position of phosphenes was evaluated in two ways: qualitatively and quantitatively. Figure 5 shows the data analysis method for qualitative evaluation. As mentioned in Section 3.1, subjects drew the position of the phosphene using a mouse with a circle tool. The number of images of the phosphene presentation position drawn by a subject was 204 in total, with 17 fixation points, four electrode arrangements, and three patterns of red–green glasses conditions. For subjects with strabismus, the position of the phosphene presentation may differ depending on whether they wear red–green glasses. However, we assumed that the position of the presented phosphene would not change for subjects with normal eye movements. Therefore, 68 patterns of phosphene presentation positions were superimposed for each subject, with 17 gaze positions and four electrode arrangements. By setting the opacity of the phosphene drawn by each subject to 10% and superimposing them, if many subjects observed phosphenes in the same area, the drawn circles would overlap and be presented in white, as shown in Figure 5. The above evaluation method showed the tendency of the phosphene presentation position to change depending on the gaze position. By showing the tendency of the phosphene presentation position for each experimental condition, the extent to which the phosphene presentation position changed with eye movement was evaluated.

**Figure 6:**
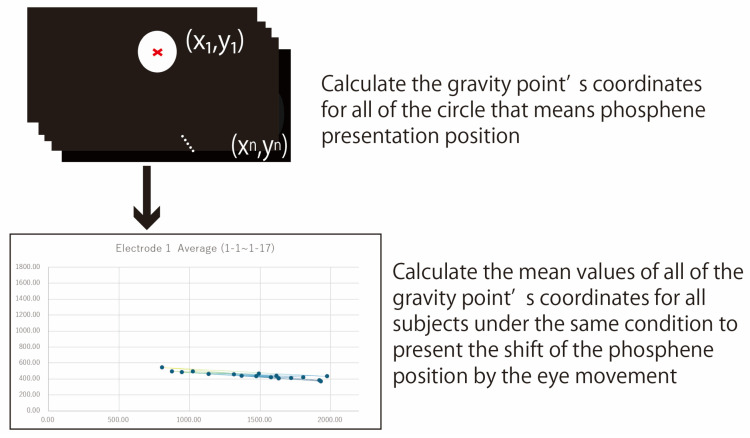
Quantitative evaluation methods.

Figure 6 shows the data analysis method for quantitative evaluation. The gravity points of the circle drawn by the subject as the phosphene presentation position were calculated, and the average of the gravity points of the 68 patterns of phosphenes for each subject with 17 fixation points and 4 electrode arrangements was calculated. The number of replicates for each data point was three (trial number of each subject) times ten (number of subjects). Therefore, each point of the graph in Figure 6 has an average value of 30 data. The gravity points of the 68 patterns of phosphenes for all subjects were calculated, and the extent to which the coordinates changed with eye movement was evaluated. The lines connecting each point show a dark blue to yellow gradation from fixation points 1 to 17, as shown in Figure 6. In the case of focusing around the central vision, the line was colored dark blue, and in the case of focusing around the peripheral vision, the line was colored yellow. The image depicting the phosphene was 2200 pixels wide, and the experiment was conducted to present phosphenes in three directions: right, center, and left.

A change in phosphene presentation position was evaluated when the change in phosphene presentation position was greater than 733 pixels, which is obtained by dividing 2200 pixels into thirds.

## 4. Verification of Experimental Results

In this section, the qualitative and quantitative evaluations of the results of the verification experiment are presented. First, the qualitative evaluation is presented.

**Figure 7:**
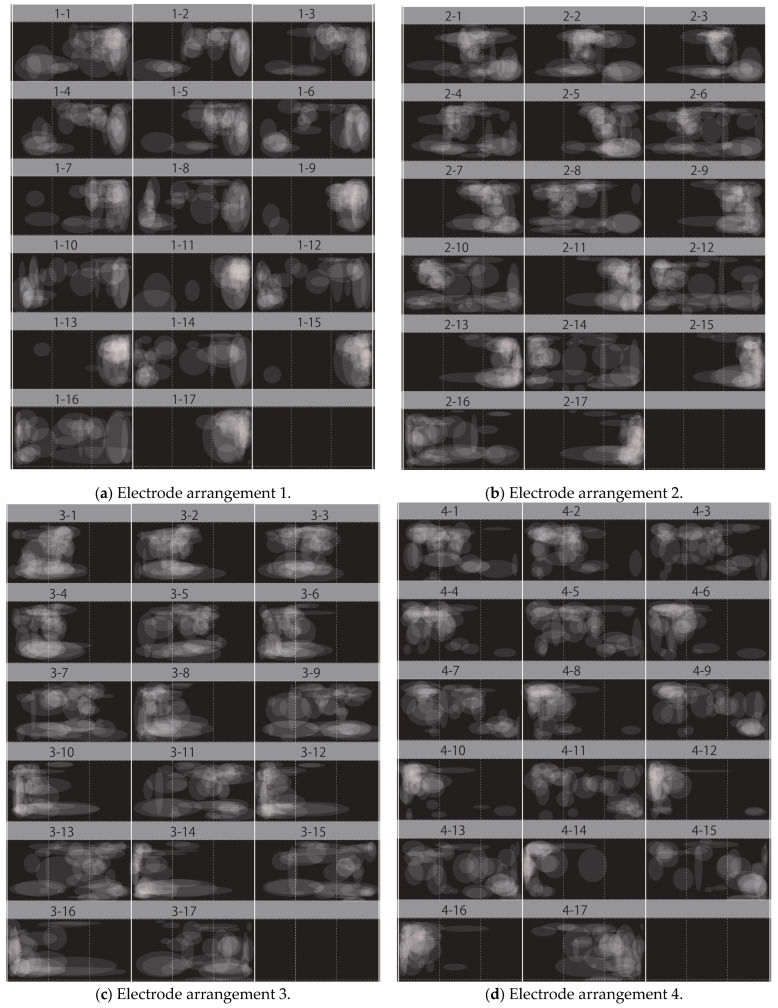
Qualitative verification experiment results. (**a**) Verification experiment results using electrode arrangement 1. (**b**) Verification experiment results using electrode arrangement 2. (**c**) Verification experiment results using electrode arrangement 3. (**d**) Verification experiment results using electrode arrangement 4.

Figure 7a shows the verification results for electrode arrangement 1, with the phosphene presentation positions of all subjects superimposed. Similarly, Figure 7b shows the results for electrode arrangement 2, Figure 7c for electrode arrangement 3, and Figure 7d for electrode arrangement 4. The numbers 1 to 17 correspond to the fixation points on the Hess chart shown in Figure 3.

Fixation points 1 to 7 represent a visual field of ±15 deg, 8 to 15 represent a visual field of ±30 deg, 16 represents the case where the eyeball was moved to the left as far as possible, and 17 represents the case where the eyeball was moved to the right as far as possible. The white circles represent the presentation positions of the phosphene drawn by the subjects, and drawing them on the right side of the image means that the phosphene was presented on the right side of the visual field.

Since Figure 7a was a verification experiment using electrode arrangement 1, phosphenes were generally presented on the right side of the visual field at fixation points 1 to 7. Some subjects appeared to observe phosphenes on the left side of the visual field, because the Ora Serrata inside the eyeball was located closer to the anterior side. The position of the retina inside the eyeball shown in Figure 1 in Section 2.1 is based on a previous study [3], but there are individual differences. Therefore, subjects who observed phosphenes on the left side of the visual field with electrode arrangement 1 would be able to observe them on the right side of the visual field by adjusting the electrode arrangement and stimulating current value. In addition, Figure 7a shows that, in the verification experiment results for electrode arrangement 1, some subjects observed phosphenes in the center of the visual field. This result also suggests that electrode arrangement 1 may have stimulated the area that was originally intended to be stimulated with electrode arrangement 2. Therefore, it is believed that phosphenes can be presented on the right side of the visual field by adjusting the electrode arrangement position of the relevant subjects. Figure 7b,c show the results of a verification experiment using electrode arrangements 2 and 3, which present phosphenes in the center of the visual field. These results show that phosphenes were presented approximately near the center of the visual field at fixation points 1 to 7. Because electrode arrangement 2 stimulates the right eyeball and electrode arrangement 3 stimulates the left eyeball, it appears that phosphenes were presented to the right of the center of the visual field when electrode arrangement 2 was used, and to the left of the center of the visual field when electrode arrangement 3 was used. This is because a previous study [4] reported that it was difficult to stimulate the area near the inner corner of the eye due to the shape of the eye socket. It is speculated that the area from the center of the stimulated eyeball to the outer corner of the eye was stimulated with electrode arrangements 2 and 3, which was reflected in the presentation position of the phosphenes.

Figure 7d shows the results of a verification experiment using electrode arrangement 4, and it can be seen that phosphenes were presented approximately on the left side of the visual field at fixation points 1 to 7. Some subjects were stimulated around the Ora Serrata as, with electrode arrangement 1, the phosphene was presented on the right side of the visual field. Some subjects were stimulated around the center of the eye, which was originally intended to be stimulated by electrode arrangement 3, as with electrode arrangement 1. Therefore, by adjusting the electrode arrangement position of the relevant subjects, phosphenes can be presented on the left side of the visual field.

With both electrode arrangements, there is no significant change in the presentation position of the phosphenes when the subjects are gazing at the central visual field. However, from fixation point 6, where eye movement exceeds 15 deg, the presentation position of the phosphenes appears to differ in each subject. In addition, it can be seen that phosphenes were generally observed to the right at odd fixation points after fixation point 8 in Figure 7a,b, which used electrode arrangements that stimulate the right eye. These results were attributed to the fact that, at odd fixation points, the retina controlling the right side of the visual field was exposed to the anterior side by moving the eye to the right and was directly stimulated. In addition, it was widely observed from the left to the center at even fixation points. The even fixation points were trials in which the eyeball was moved to the left, and as mentioned in Section 2.1, the retina on the temporal side of the right eye controls the left side of the visual field but the nose blocks the view, and so the peripheral vision in the left visual field cannot be recognized. This makes it difficult to recognize how far the phosphenes moved to the left of the visual field, and it is thought that they were widely observed from the left to the center.

It can be seen that phosphenes were generally observed to the left at even fixation points after fixation point 8 in Figure 7c,d, which used electrode arrangements that stimulated the left eye. These results are because, at even fixation points, the retina controlling the left side of the visual field was exposed to the anterior side by moving the eyeball to the left and was directly stimulated. Additionally, it was widely observed from the right to the center at odd fixation points. As with the right eye, the visual retina on the temporal side of the left eye controls the right side of the visual field, but because the nose blocks the field of vision, it is unable to recognize the peripheral vision on the right side of the visual field. This makes it difficult to recognize how far the phosphenes moved to the right of the visual field, and it is thought that they were observed widely from the right to the center.

**Figure 8:**
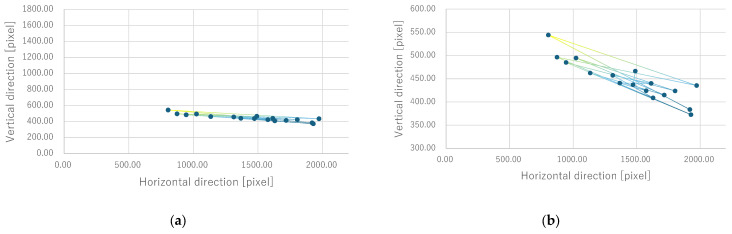
Quantitative experimental results with electrode arrangement 1 at all fixation points. (**a**) Experiment results with image size drawn by subjects. (**b**) Experiment results with the size of the image drawn by the subject, enlarged on the y-axis.

**Figure 9:**
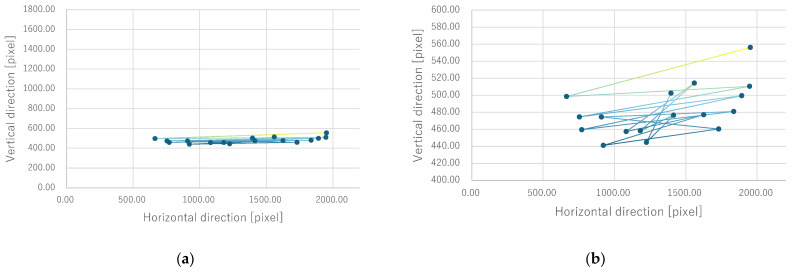
Quantitative experimental results with electrode arrangement 2 at all fixation points. (**a**) Experiment results with image size drawn by subjects. (**b**) Experiment results with the size of the image drawn by the subject, enlarged on the y-axis.

**Figure 10:**
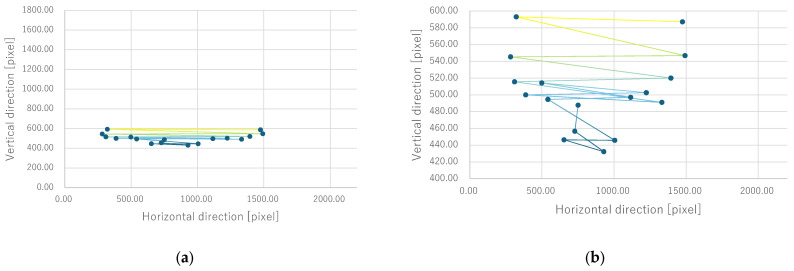
Quantitative experimental results with electrode arrangement 3 at all fixation points. (**a**) Experiment results with image size drawn by subjects. (**b**) Experiment results with the size of the image drawn by the subject, enlarged on the y-axis.

**Figure 11:**
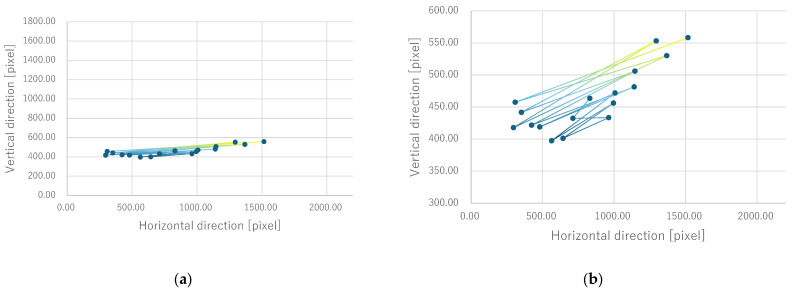
Quantitative experimental results with electrode arrangement 4 at all fixation points. (**a**) Experiment results with image size drawn by subjects. (**b**) Experiment results with the size of the image drawn by the subject, enlarged on the y-axis.

Next, the quantitative evaluation of the result of the verification experiment is mentioned. Figure 8 shows the average of the gravity point coordinates of the circles, indicating the presentation position of phosphenes drawn by all subjects when electrode arrangement 1 was used. Similarly, Figure 9, Figure 10 and Figure 11 show the average gravity point coordinates of the phosphene presentation positions for electrode arrangements 2, 3, and 4, respectively. In Figure 8a, Figure 9a, Figure 10a and Figure 11a, the horizontal and vertical axes show the size of the images drawn by the subjects, indicating the phosphene presentation positions, 0 to 2200 pixels and 0 to 1800 pixels, respectively. In Figure 8b, Figure 9b, Figure 10b and Figure 11b, the vertical axes were enlarged from the images drawn by the subjects to 0 to 600 pixels, indicating the phosphene presentation positions. Each point shows the average phosphene presentation positions at each fixation point, and the average phosphene presentation positions for fixation points 1 to 17 are connected by lines in numerical order. The lines connecting each point show a dark blue to yellow gradation from fixation points 1 to 17.

**Figure 12:**
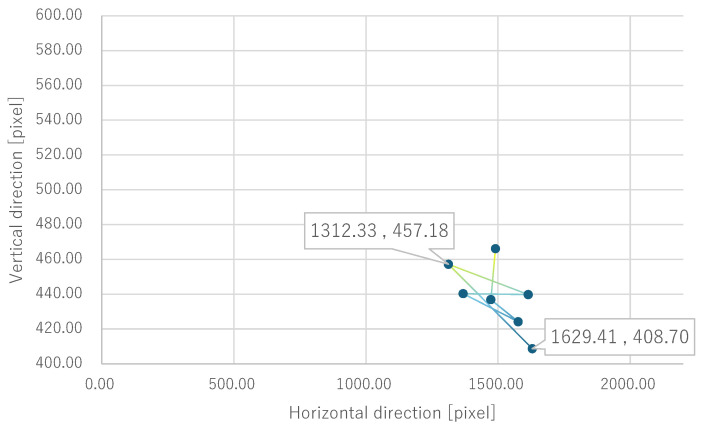
Quantitative experimental results with electrode arrangement 1 within a range of ±15 deg of central vision.

**Figure 13:**
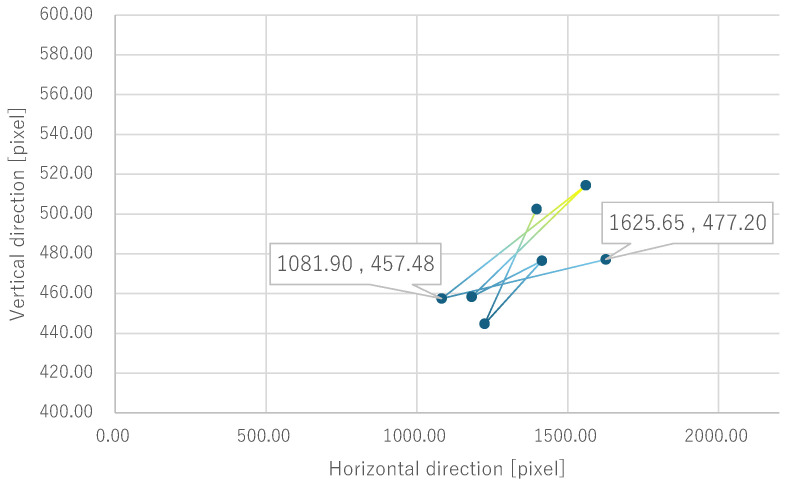
Quantitative experimental results with electrode arrangement 2 within a range of ±15 deg of central vision.

**Figure 14:**
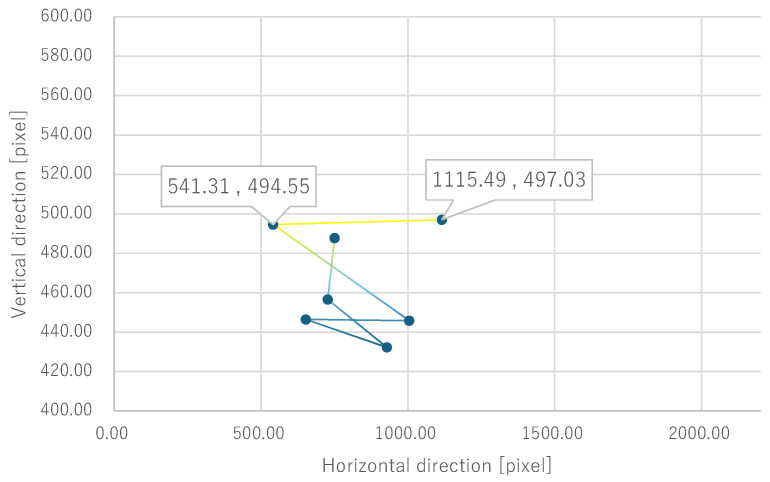
Quantitative experimental results with electrode arrangement 3 within a range of ±15 deg of central vision.

**Figure 15:**
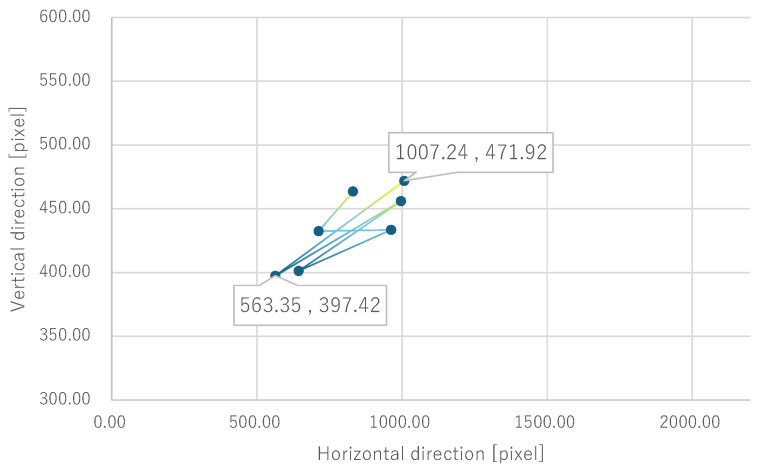
Quantitative experimental results with electrode arrangement 4 within a range of ±15 deg of central vision.

For all electrode arrangements, the phosphene presentation positions also fluctuate significantly as the fixation point moves toward the peripheral visual field. Figure 12, Figure 13, Figure 14 and Figure 15 show the average phosphene presentation positions at fixation points 1 to 7 when electrode arrangements 1, 2, 3, and 4 were used, respectively. Comparing Figure 8 and Figure 12, the fluctuation in the presentation position of the phosphene has become smaller. Similarly, comparing Figure 9 and Figure 13, Figure 10 and Figure 14, and Figure 11 and Figure 15, it is clear that the fluctuation in the presentation position of the phosphene has become smaller. Therefore, it was shown that the fluctuation in the presentation position of the phosphene is small within the range of eye movement of ±15 deg. The right and left ends of the phosphene presentation position within a range of ±15 deg of eye movement are highlighted in Figure 12, Figure 13, Figure 14 and Figure 15. The x-coordinates of these highlighted points were used to calculate the range of phosphene fluctuation. When eye movement was within a range of ±15 deg, the fluctuation in the phosphenes’ presentation position was insignificant. The difference in the horizontal axis fluctuation was 317.08 pixels for electrode arrangement 1, 543.75 pixels for electrode arrangement 2, 574.18 pixels for electrode arrangement 3, and 443.89 pixels for electrode arrangement 4. In Section 3.2, it was mentioned that, if the fluctuation in the presentation position of the phosphenes is greater than 733 pixels, which is 2200 pixels divided into thirds, it can be interpreted as a significant change in the presentation position of the phosphenes. It was found that, for all electrode arrangements, when eye movement was within a range of ±15 deg, the fluctuation was smaller than 733 pixels. Therefore, when eye movement was within a range of ±15° for all electrode arrangements, the phosphenes did not show a significant change in the presentation position.

## 5. Discussion

This section discusses the position of phosphenes for subjects in whom phosphenes were presented in an unintended direction in the results of the verification experiments. The effect on the transition of the phosphene presentation position for all subjects, except those who observed phosphenes in an unintended direction, is also shown by excluding the results of these specific subjects. In addition, the safety of a walking assistive device using phosphenes for visually impaired people is discussed, taking into account the internal structure of the eyeball described in Section 2.1, the angle of eye movement during walking reported in the related study described in Section 2.2, and the change in the presentation position of phosphenes due to eye movement obtained in the verification experiment of this study.

Table 1 shows the presentation position of phosphenes observed by the subjects when using electrode arrangements 1 and 4. Electrode arrangement 1 was used to present phosphenes to the right side of the visual field, and electrode arrangement 4 was used to present phosphenes to the left side of the visual field. However, a previous study has stated that unintended areas may be stimulated depending on the shape of the subject’s face [4]. The electrode arrangement used in this study was based on the electrode arrangement reported in previous studies [4]. However, the electrode arrangements were not calibrated for each subject in this experiment. As a result, it is likely that some subjects experienced unintended phosphene presentations, as shown in Table 1.

**Figure 16:**
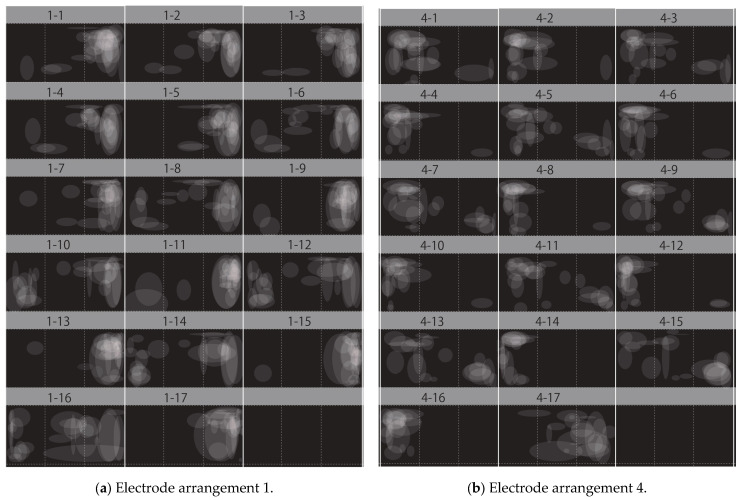
Qualitative results of the verification experiment results with only those subjects who presented the phosphenes at the intended position. (**a**) Verification experiment results using electrode arrangement 1. (**b**) Verification experiment results using electrode arrangement 4.

Figure 16a shows the transition of the phosphene presentation position for all sub-jects, excluding the results of the subjects who did not experience phosphene presentation to the right of the visual field when using electrode arrangement 1, as shown in Table 1. Similarly, Figure 16b shows the transition of the phosphene presentation position for all subjects, excluding the results of the subjects who did not experience phosphene presentation to the left of the visual field when using electrode arrangement 4, shown in Table 1. At fixation points 1 to 7, it can be seen that the phosphene is presented to the right in Figure 16a and the left in Figure 16b. As shown in Table 1, some subjects experienced phosphene presentation not only to the right of the visual field when using electrode arrangement 1 but also in the center or left side. Similarly, when electrode arrangement 4 was used, some subjects observed phosphenes not only on the left side of the field of view but also on the center or right side. Thus, Figure 16a shows that phosphenes were presented in the center and left side of the visual field, but the number of such phosphenes is clearly smaller than that in Figure 7a, which is the verification result. Similarly, in Figure 16b, phosphenes are seen to be presented in the center and right side of the visual field, but the number of such phosphenes is clearly smaller than that in Figure 7d, which is the verification result.

**Figure 17:**
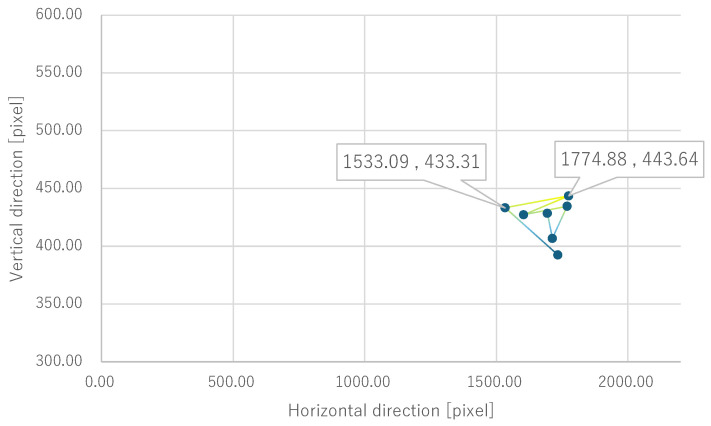
Quantitative experimental results with only the subjects who presented the phosphenes at the intended position with electrode arrangement 1 within a range of ±15 deg of central vision.

**Figure 18:**
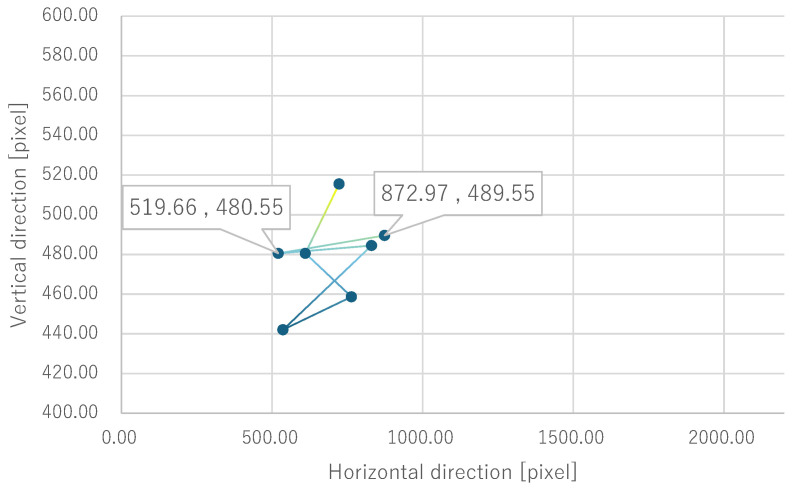
Quantitative experimental results with only the subjects who presented the phosphenes at the intended position with electrode arrangement 4 within a range of ±15 deg of central vision.

Furthermore, under the same subject conditions, the average of the gravity points of the presentation positions of the phosphenes drawn by the subjects was calculated, and only the range of fixation points 1 to 7 is shown in Figure 17 for electrode arrangement 1 and Figure 18 for electrode arrangement 4. The extent to which the presentation position of the phosphenes does not fluctuate due to eye movement is discussed in comparison to the verification results.

Comparing Figure 12 and Figure 17 when electrode arrangement 1 was used, the distribution shown in Figure 17 was to the right of the average gravity points of the phosphene presentation positions shown in Figure 12. In Figure 17, the fluctuation of the phosphene presentation positions was less than that in Figure 12. The difference in the horizontal axis fluctuation was 317.08 pixels in Figure 12; however, it was clearly smaller at 241.79 pixels in Figure 17. Similarly, comparing Figure 15 and Figure 18 when electrode arrangement 4 was used, the distribution shown in Figure 18 is to the left of the average center of gravity of the phosphene presentation positions shown in Figure 15. In Figure 18, the fluctuation of the phosphene presentation positions is smaller than that in Figure 15, and the difference in the horizontal axis fluctuation was 443.89 pixels in Figure 15; however, it was clearly smaller at 353.31 pixels in Figure 18. In Section 3.2, it was mentioned that, if the fluctuation in the presentation position of the phosphenes is greater than 733 pixels, which is 2200 pixels divided into thirds, it can be interpreted as a significant change in the presentation position of the phosphenes. Therefore, it was suggested that, if the optimal electrode arrangement for the subject was selected so that the phosphene could be presented in the intended direction, the fluctuation of the phosphene presentation position within the range of eye movement of ±15 deg would not be a safety issue.

Considering the results of the related study on eye movements during walking, as shown in Section 2.2, the safety of the walking assistive device for the visually impaired using phosphenes is discussed.

The assistive device in this study was aimed at visually impaired people with peripheral damage to the visual pathway, including those with retinitis pigmentosa, which was the subject of the related study [13] mentioned in Section 2.2. The reason why retinitis pigmentosa patients can be targeted in this study is that electrical stimulation from electrodes placed on the face stimulates the retinal ganglion cells and amacrine cells, but not the rod and cone cells that are damaged by retinitis pigmentosa, except when the retinal photoreceptors are exposed to the anterior side due to eye movement [3,17,18].

This suggests that the current flowing through the anterior side stimulates ganglion cells and amacrine cells, and it is possible to present phosphenes by ocular stimulation to patients with retinitis pigmentosa.

As mentioned in Section 2.2, the eye movement of patients with retinitis pigmentosa during walking was within 2/3 of their normal visual field [13]. In addition, in experiments with sighted subjects, eye movement during walking were generally considered to be within 15 deg [12].

When considering this information and the actual phosphene presentation position during eye movement reported in the verification experiment of this study, it can be concluded that the presentation position of the phosphene does not change significantly within the range of eye movement during walking reported in related studies [12,13], and that this result does not threaten the safety of walking assistive devices for visually im-paired people using phosphenes.

In addition, as shown in Section 2.1, the angles required for the right eye to expose the Ora Serrata on the anterior side through eye movement are 77π/300 rad and 17π/60 rad on the left and right, respectively. In degrees, these are 18.6 deg and 11 deg, respectively. An angle of 11 deg is a small eye movement compared to the ±15 deg that was the focus of this study, and if the change in the presentation position of the phosphene due to the anterior exposure of the Ora Serrata was significant, the safety of the phosphene walking assistive device would be impaired. For the Ora Serrata when looking at the presentation position of phosphene drawn by the subject in Figure 16, there is variation at fixation points 4 and 6 when using electrode arrangement 1 compared to fixation points 1, 2, and 3. However, as mentioned above in this section, when looking at the average value of the gravity points of the presentation position of phosphene shown in Figure 17 and Figure 18, it is clear that the variation is within a range that does not cause problems under the premise of presenting the phosphene in three directions of the visual field.

Because even if the Ora Serrata is exposed to the anterior side by moving the eyeball 11deg, there are few retinal photoreceptors distributed near the Ora Serrata, and the stimulus is not effectively delivered.

From the above, the effect of the change in the presentation position of phosphene due to eye movement during walking on the safety of the walking assistive device for visually impaired people using phosphenes is extremely small.

## 6. Conclusions

In this study, the change in the presentation position of phosphenes was investigated when eye movements were performed gradually, and the extent of the eye movement led to a large change in phosphenes. It was demonstrated that a change in the presentation position of phosphenes during eye movements in the range of ±15 deg does not affect the safety of the walking assistive device for visually impaired people using phosphenes.

The walking assistive device for visually impaired people using phosphenes that was the subject of this study stimulates the eyeball; so, it can only be used by patients with peripheral damage to the visual pathway and cannot be used by patients with damage to the optic nerve, etc. In the future, it will be necessary to investigate the presentation method of phosphenes that stimulate the visual cortex to make it applicable to a wider range of visually impaired people.

## Figures and Tables

**Table 1:** The presentation position of phosphenes observed when using electrode arrangements 1 and 4.

	Phosphene Presentation	Subject 1	Subject 2	Subject 3	Subject 4	Subject 5	Subject 6	Subject 7	Subject 8	Subject 9	Subject 10
Electrode 1	Presented phosphenes at right side	〇 *	〇	〇	×	〇	〇	×	〇	×	〇
	Presented phosphenes at left side	×	〇	×	〇	×	×	×	×	×	×
	Presented phosphenes at central side	×	×	×	×	〇	×	〇	×	〇	〇
	Phosphenes did not move to the left side during eyeball movement	〇	×	〇	×	×	×	〇	〇	×	×
Electrode 2	Presented phosphenes at left side	〇	〇	×	〇	×	〇	×	〇		×
	Presented phosphenes at right side	〇	×	〇	〇	×	×	×	×	×	×
	Presented phosphenes at central side	×	〇	〇	×	〇	×	〇	×	〇	〇
	Phosphenes did not move to the right side during eyeball movement	〇	×	〇	×	×	〇	〇	〇	×	×

* “〇” means that the subject observed phosphenes in this condition. “×” means that the subject did not observe phosphenes in this condition.

## Data Availability

The raw data supporting the conclusions of this article will be made available by the authors on request.

## Abbreviations

The following abbreviations are used in this manuscript:

tACS	Transcranial alternating current stimulation
EOG	Electrooculogram
